# Matrix Metalloproteinases, Tissue Inhibitors of Metalloproteinases, and Their Ratios in Women with Polycystic Ovary Syndrome and Healthy Controls

**DOI:** 10.3390/ijms26010321

**Published:** 2025-01-01

**Authors:** Alexandra E. Butler, Manjula Nandakumar, Thozhukat Sathyapalan, Edwina Brennan, Stephen L. Atkin

**Affiliations:** 1 Research Department, Royal College of Surgeons of Ireland, Adliya 15503, Bahrain; mnandakumar@rcsi.com (M.N.); ebrennan@rcsi.com (E.B.); satkin@rcsi.com (S.L.A.); 2 Academic Endocrinology, Diabetes and Metabolism, Hull York Medical School, Hull HU6 7RX, UK; thozhukat.sathyapalan@hyms.ac.uk

**Keywords:** polycystic ovary syndrome, matrix metalloproteinases, tissue inhibitors of metalloproteinases, A disintegrin and metalloproteinase with thrombospondin motifs

## Abstract

Matrix metalloproteinases (MMPs) are M2 macrophage markers that are modulated by inflammation. A disintegrin and metalloproteinases (ADAMS) and those with thrombospondin motifs (ADAMTS) regulate the shedding of membrane-bound proteins, growth factors, cytokines, ligands, and receptors; MMPs, ADAMS, and ADAMTS may be regulated by tissue inhibitors of metalloproteinases (TIMPs). This study aimed to determine whether these interacting proteins were dysregulated in PCOS. A Somascan proteomic analysis of 12 MMPs, three of their inhibitors (TIMP-1, 2, 3), two ADAMS (9, 12), five ADAMTS (1, 4, 5, 13, 15), insulin-like growth factor binding protein-1 (IGFBP-1), and insulin-like growth factor-1 (IGF-1) was undertaken in a well-validated PCOS database of 143 women with PCOS and 97 controls. Women with PCOS had significantly higher levels of MMP-9 and lower levels of MMP-2, MMP-14, TIMP-2, IGFBP-1, and IGF-1 compared to the controls (*p* < 0.0001, *p* < 0.005, *p* < 0.04, *p* < 0.05, *p* < 0.0001, and *p* < 0.0001, respectively). No differences were observed for any other MMPs. The ADAMS or ADAMTS levels did not differ between groups. Body mass index (BMI) was correlated with MMP-9 (*p* < 0.01), MMP-1 (*p* < 0.05), MMP-2 (*p* < 0.05), MMP-10 (*p* < 0.005), MMP-12 (*p* < 0.005), ADAM-9 (*p* < 0.05), and IGFBP-1 (*p* < 0.0001), but only MMP-9 still differed after accounting for BMI. MMP-9/TIMP-1, MMP-9/TIMP-2, and MMP-9/TIMP-3 ratios were higher in the PCOS group (*p* < 0.01), whilst MMP-17/TIMP-1 and MMP-17/TIMP-2 were lower (*p* = 0.01). MMP-2/TIMP ratios showed no difference between groups. TIMP-2 was positively correlated with CRP (*p* < 0.01). MMP changes in PCOS are largely driven by BMI, though increased MMP-9 is BMI-independent, suggesting that any deleterious effects of MMP-9 would be potentially exacerbated by a concomitantly increased BMI. The significant increases in the MMP-9/TIMP ratios suggests MMP-9 overactivity in PCOS.

## 1. Introduction

The metzincin superfamily of metalloproteinases are a group of proteolytic enzymes which possess zinc ion-binding methionine-turn sequences and include matrix metalloproteinases (MMPs), A disintegrin and metalloproteinases (ADAMs), and A disintegrin and metalloproteinases with thrombospondin motifs (ADAMTSs) [1]. MMPs are calcium-dependent proteases, both secretory and membrane bound, comprising 23 members classified based on substrate specificity and their function as collagenases, gelatinases, stromelysins, matrilysins, membrane-type (MT)-MMPs, and other non-classified MMPs [2,3]. MMPs are produced in various cell types, including epithelial cells, fibroblasts, and inflammatory cells [4]. MMPs are responsible for remodelling the extracellular matrix (ECM) [3,5] and also play a role in activating receptors, growth factors, cytokines, adhesion molecules, and other pericellular proteins at the cell surface and in processing various signalling molecules [6,7]. MMPs are regulated at the transcriptional level with external factors such as ECM molecules, hormones, and growth factors (transforming growth factor beta (TGF-β), epidermal growth factor receptor (EGFR), tumour necrosis factor (TNF)-α, and interleukin (IL)-1β), impacting gene expression, and they are converted to their active forms through zymogen activation [8]. Thirteen ADAMs are membrane anchored, playing a role in the extracellular domain shedding of membrane-anchored proteins, cytokines, growth factors, ligands, and their receptors, while nineteen ADAMTSs are secretory, cleaving non-fibrillar ECM proteins [9]. Activated MMPs, ADAMs, and ADAMTSs are inhibited by four tissue-specific endogenous secretory tissue inhibitors of metalloproteinases (TIMPs).

**Figure 1:**
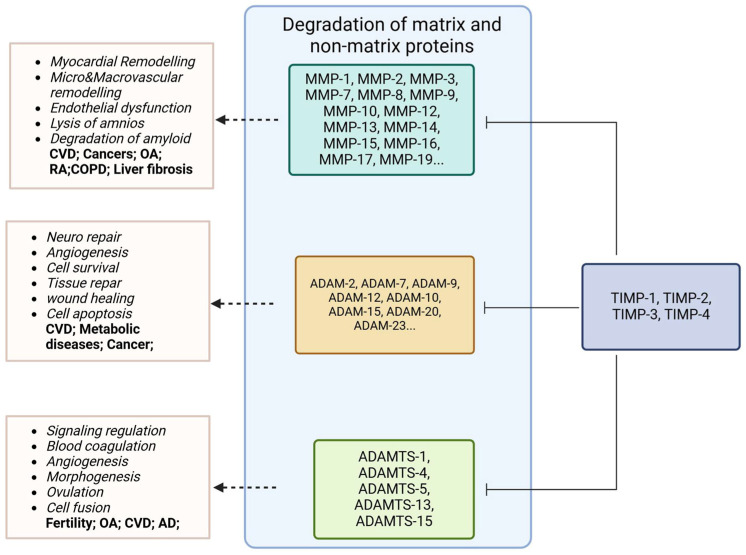
An illustration of matrix metalloproteinases (MMPs), A disintegrin and metalloproteinases (ADAMs), and A disintegrin and metalloproteinases with thrombospondin motifs (ADAMTS), which share the common function of degrading matrix and non-matrix proteins. Their key pathophysiological roles and the disease outcomes are highlighted. These functions are inhibited by the tissue inhibitors of metalloproteinases (TIMPS). MMP—matrix metalloproteinase; ADAM—A disintegrin and metalloproteinase; ADAMTS—A disintegrin and metalloproteinase with thrombospondin motifs; TIMP—tissue inhibitor of metalloproteinase; CVD—cardiovascular diseases; OA—osteoarthritis; RA—rheumatoid arthritis; AD—Alzheimer’s disease; COPD—chronic obstructive pulmonary disease.

The diversity of metalloproteinases and their physiological substrates determines whether TIMPs inhibit matrix proteolysis or accumulation [10]. TIMPs can target multiple metalloproteinases, and each metalloproteinase can recognise multiple protein substrates. Metalloproteases, together with their inhibiting TIMPs, are capable regulating signalling downstream of TNF and IL6 receptors, important modulators of inflammatory processes of the immune response system [11]. Consequently, the TIMP–metalloproteinase–substrate network controls a powerful cascade inherent to normal tissue homeostasis and physiological processes [1]. Imbalances in the levels and activity of this network and its involvement in inflammatory processes have been linked with various pathological conditions, including cancer, cardiovascular disease, peripheral vascular disease, inflammatory disease, and metabolic disease [9,12,13,14,15,16,17]. In fact, the ratio of MMPs to the inhibitory TIMP reflects the relative activity of the underlying ECM modelling, and these ratios have been suggested as biomarkers in different disease states [18]. Figure 1 shows the interaction of the MMPs, ADAMS, ADAMTS, and TIMPs, highlighting their common ECM and non-ECM protein substrates in addition to some of their pathophysiological roles and the disease outcomes they have been associated with.

In the ovaries, periodic ECM remodelling and angiogenesis are essential for follicular development and maturation, ovulation, and the establishment of the luteal corpus. This is coordinated by various proteins, steroids, energy metabolites, cytokines, and growth factors such as TGF-β [19]. Remodelling of the ECM is largely achieved by metalloproteinases, such as collagenases and gelatinases (MMP-2 and MMP-9), under the regulation of their TIMPs [20]. MMPs, as well as their endogenous inhibitors, TIMPs, have been implicated in ovarian physiology (follicular rupture and oocyte release) as well as ovarian pathophysiology [21]. It is therefore not surprising that the TIMP–metalloproteinase–substrate network appears to be involved in the pathogenesis of polycystic ovary syndrome (PCOS) [22,23,24].

PCOS is the most common endocrine disorder among women of reproductive age with a reported prevalence of 6–10% [25], with incidence rates increasing in younger age groups [26]. Menstrual irregularity, anovulatory infertility, and hirsutism are classic features of PCOS. An imbalance between MMPs and TIMPs, with lower concentrations of TIMP-2 and increases in MMP-2/TIMP-2 and MMP-9/TIMP-1 ratios, has been reported in PCOS [27]; however, there remains controversy as to whether MMP-2 and/or MMP-9, for example, are elevated [23,24,28], unchanged [27,29], or reduced [30] in PCOS, where they have been shown to correlate with BMI but with their significance being lost when BMI was accounted for [31].

MMPs and ADAM/ADAMTS share a common function in regulating tissue remodelling through the degradation of matrix or non-matrix proteins. ADAMTS perform several functions, including vascular smooth muscle cell proliferation and migration, angiogenesis, vascular cell apoptosis, cell survival, tissue repair, and wound healing [32]. The expression level of ADAMTS-1 was positively correlated with the oocyte maturation rate and good-quality embryo rate in patients with PCOS [33], and it has been shown that the dysregulated expression of ADAMTS-1 in PCOS may influence oocytes though granulosa cells [34]. ADAMTS-1 has also been shown to positively correlate with versican, a large hyaluronic acid-binding proteoglycan expressed by periovulatory granulosa cells, in patients with PCOS, suggesting that ADAMTS-1 has a role in ovulatory dysfunction and in the pathogenesis of PCOS [35]. A recent systematic review and meta-analysis found that ADAMTS-1, ADAMTS-4, ADAMTS-5, and ADAMTS9 downregulation were negatively associated with various in vitro fertilisation and embryo transfer outcomes in women with PCOS, including elevated follicle puncture [36].

In PCOS, there is also a recognised increase in the prevalence of metabolic features, including type 2 diabetes (T2D), hypertension, and cardiovascular disease [37]. Although the inherent mechanisms underlying this are still unclear, insulin resistance (IR) and obesity-related inflammation associated with PCOS have been implicated [37,38]. The availability of free insulin-like growth factor-1 (IGF-1) and its tissue action is dependent on its binding to insulin-like growth factor binding proteins (IGFBPs) [39]. MMPs have been shown to cleave IGFBP-1, whose levels are decreased in PCOS [23]. Indeed, MMP-7 has been shown to cleave all six IGFBPs, leading to IGF-1 bioavailability and phosphorylation of the IGF-1 receptor [39], resulting in higher levels of IGF-1. IGF-1 is well recognised as a tumour-promoting growth factor and is involved in tumour metastases, but it is recognised as being integral in extracellular tissue modelling through increasing collagen metabolism and fibroblast growth [40].

Based on the above, we hypothesised that, when obesity and IR were accounted for in a robust cohort, the metalloproteinase changes seen would be inherent to PCOS. In this study, we analysed plasma to detect any differences in MMPs, ADAMS, ADAMTS, and TIMP from women with and without PCOS from a UK Biobank. We also examined MMP/TIMP ratios as proposed biomarkers for PCOS.

## 2. Results

### 2.1. Demographics

The 137 subjects with PCOS and 97 controls were age-matched, but the subjects with PCOS had a greater BMI (33 versus 25 kg/m^2^; *p* < 0.0001), increased HOMA-IR (2.6 versus 1.3; *p* < 0.005), and increased C-reactive protein (CRP, an inflammatory marker) (3.1 versus 1.0 mg/L; *p* = 0.0008). The subjects with PCOS had increased hyperandrogenaemia compared to the controls, which was reflected by increased testosterone (1.4 versus 1.0 nmol/L; *p* < 0.0001) and lowered SHBG (21 versus 53.5 nmol/L; *p* = 0.0003).

### 2.2. Somascan Analysis

The results of the Somascan analysis of MMP, ADAMS, ADAMTS, TIMP, IGFBP-1, and IGF-1 proteins are shown in Table 1 for the subjects with PCOS and control women. MMP-9 was increased in the subjects with PCOS compared to the controls (29,187 versus 22,064 RFU; *p* < 0.0001). MMP-2, MMP-14, TIMP-2, IGFBP-1, and IGF-1 were decreased in the subjects with PCOS compared to the controls (*p* < 0.005, *p* < 0.04, *p* < 0.048, *p* < 0.0001, and *p* < 0.0001, respectively). There were no differences in the protein levels of ADAMs or ADAMTS in the subjects with PCOS and controls (*p* > 0.05).

A comparison of the MMP/TIMP ratios between the subjects with PCOS and control subjects is shown in Table 2. The MMP-9/TIMP-1, MMP-9/TIMP-2, and MMP-9/TIMP-3 ratios were all higher in the subjects with PCOS (*p* < 0.01), as were the MMP-17/TIMP-1 and MMP-17/TIMP-2 ratios (*p* = 0.01). All of the MMP/ADAMS/ADAMTS to TIMP ratios were computed, but none differed other than those shown in Table 2.

### 2.3. Correlation and Covariance Analysis

**Figure 2:**
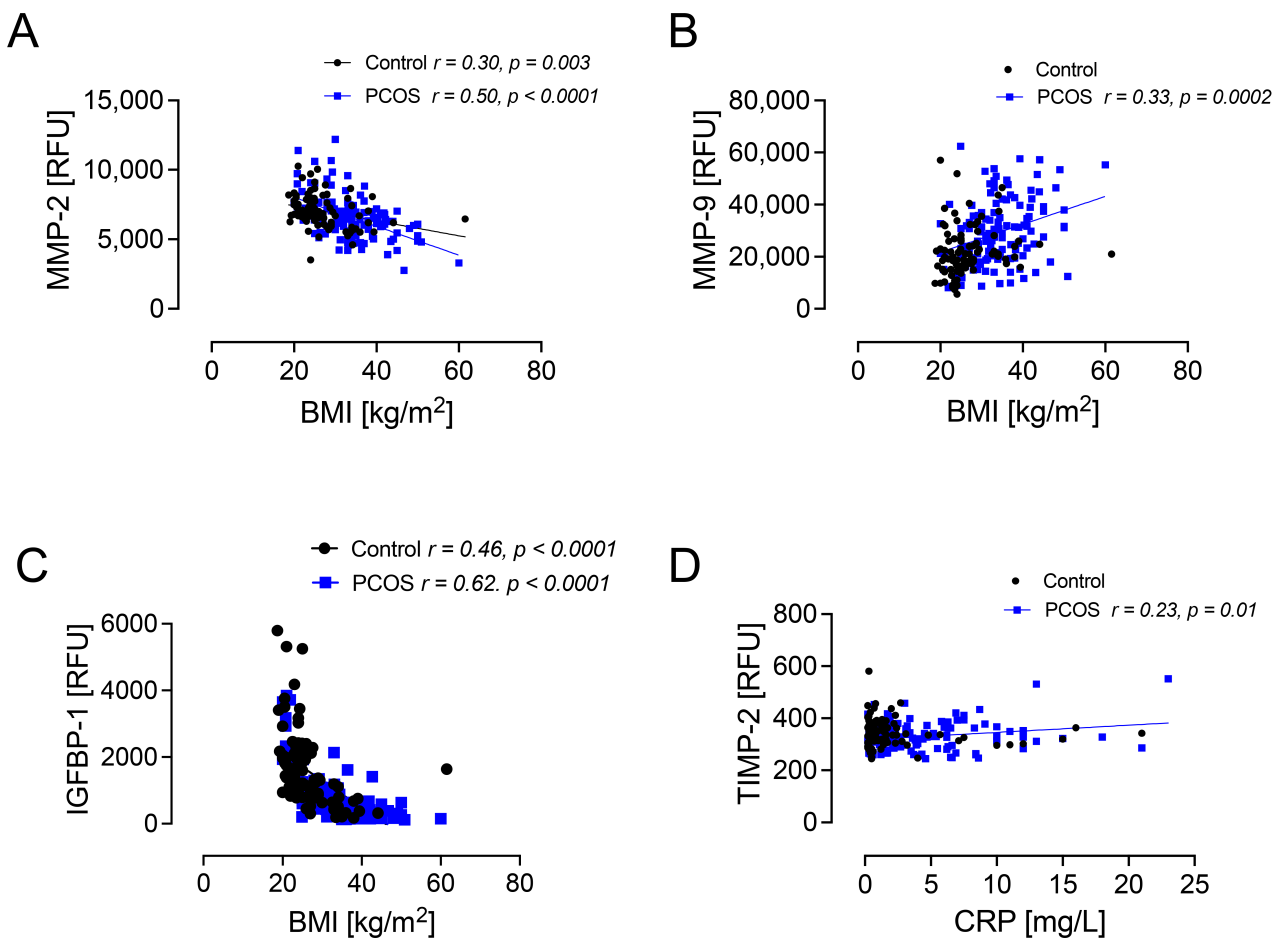
Protein scatterplots in subjects with PCOS and controls. BMI correlated negatively with MMP-2 in both controls and subjects with PCOS (**A**) and positively with MMP-9 in subjects with PCOS alone (**B**). IGFBP-1 correlated negatively with BMI in both controls and subjects with PCOS (**C**). CRP correlated positively with TIMP-2 in subjects with PCOS (**D**). MMP—matrix metalloproteinase; TIMP—tissue inhibitor of metalloproteinase; IGFBP-1—insulin-like growth factor binding protein 1; BMI—body mass index; CRP—C-reactive protein.

BMI was positively correlated with MMP-9 (*p* < 0.001) and MMP-1 (*p* < 0.01) and negatively correlated with MMP-2 (*p* < 0.0001), MMP-10 (*p* < 0.001), MMP-12 (*p* < 0.005), ADAM-9 (*p* < 0.05), ADAMTS-5 (*p* < 0.05), and IGFBP-1 (*p* < 0.0001) in subjects with PCOS, as shown in Table 3 and Figure 2. CRP was positively correlated with TIMP-2 alone in PCOS (r = 0.23; *p* < 0.01) (Figure 2). There was no correlation between HOMA-IR and MMPs, ADAMS, ADAMTS, or TIMPs. There was no correlation between testosterone and MMPs, ADAMS, ADAMTS, or TIMPs.

When an ANCOVA was undertaken and BMI was accounted for, only MMP-9 differed between the subjects with PCOS and controls (F = 5.9; *p* < 0.02).

**Figure 3:**
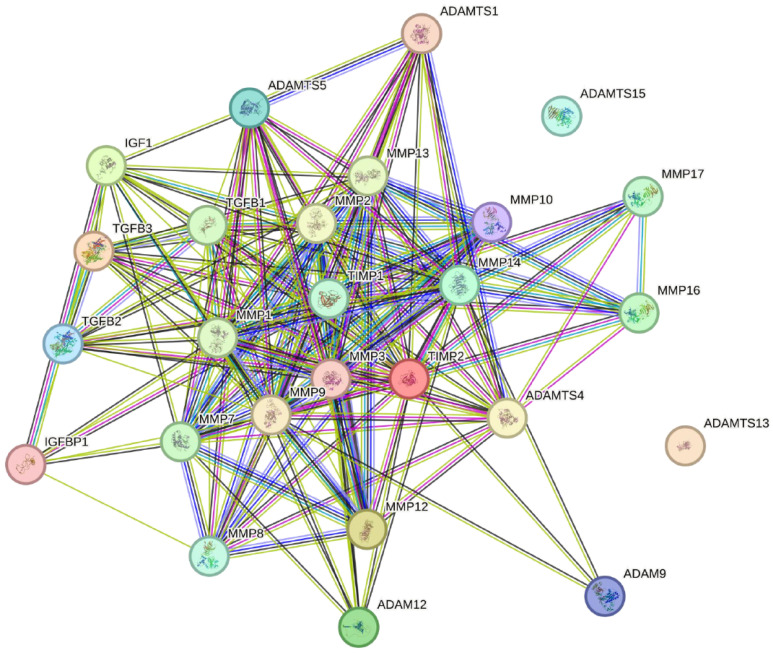
An illustration of the interactions between MMPs, ADAMs, ADAMTS, and TIMPS (STRING 12.0 application). MMP—matrix metalloproteinase; ADAM—A disintegrin and metalloproteinase; ADAMTS—A disintegrin and metalloproteinase with thrombospondin motifs; TIMP—tissue inhibitor of metalloproteinase.

STRING 12.0 (Search Tool for the Retrieval of Interacting Genes) was used to visualise the known and predicted protein–protein interactions between the MMPs, ADAMs, ADAMTS, TIMPS, IGF-1, and IGFBP-1, and they are shown in Figure 3.

## 3. Discussion

PCOS typically presents with hyperandrogenism, ovarian dysfunction, chronic oligo-anovulation, and/or polycystic morphology of the ovary. PCOS includes various phenotypes, and the pathogenesis is multifactorial, often involving insulin resistance, and is associated with several metabolic diseases including diabetes [38]. Of the four PCOS phenotypes, three are characterised with PCOM, where the recruitment of additional primordial follicles, while lacking the dominant follicle, results in ovarian enlargement, capsular thickening, and thecal/stromal hyperplasia and luteinization, which are typical of PCOM [41]. The dynamic remodelling of the ECM is integral to cell–cell interactions and signalling events required for normal folliculogenesis [42] and corpus luteum formation for progesterone production during pregnancy [43]. During normal ovarian function, changes in the production and degradation of ECM associated with altered MMP and TIMP expression and activity have been observed, which differ in PCOS.

In this study, it was shown that MMP-9 was elevated in PCOS and independently of BMI, while MMP-2 was shown to be reduced in the PCOS cohort and was BMI-dependent, as was TIMP-2. Furthermore, there were increases in the MMP-9/TIMP-1, MMP-9/TIMP-2, and MMP-9/TIMP-3 ratios in PCOS, suggesting that the increase in MMP-9 may either be due to increased levels per se or, as seen for TIMP-2, due to a reduction in the inhibitor protein. Whilst the only protein change independent of BMI was MMP-9, other protein changes were BMI-dependent, suggesting that any deleterious effects resulting from a raised MMP-9 would be potentially exaggerated by an increasing BMI.

Collagenolysis and elastolysis by MMPs occur in development, wound healing, and in major inflammatory diseases, with the MMPs proposed to be elastolytic being MMP-2, MMP-7, MMP-9, and MMP-12 [44]. However, only MMP-2 and MMP-9, both gelatinases, differed significantly in PCOS; both have overlapping and unique functions and both effectively cleave denatured collagens (gelatins) and degrade collagen types IV-V and elastin.

Collagen types VII, X, and XI; fibronectin; laminin; and non-ECM molecules are additional substrates for MMP-2, while MMP-9 degrades type III collagens, N-telopeptides of type I collagen, aggrecan, and cartilage link protein [45]. MMP-9 also activates various functional proteins, such as cytokines and TGF-β, and converts plasminogen into angiostatin [45,46]. At the transcription level of regulation, MMP-2 and MMP-9 are known to be regulated by the nuclear factor-kappa B (NF-κB) and mitogen-activated protein kinases/extracellular-signal-regulated kinases (MAPKs/ERKs) [47], while TIMPs inhibit stoichiometrically active MMPs in a 1:1 molar ratio [45].

Both MMP-9 and MMP-2 are often increased together, but there are inconsistencies in the literature. In cultured human luteinized granulosa cells following ovarian stimulation, increased expression of MMP-2 and MMP-9 was observed in PCOS, as in follicular fluid levels [48]. The same authors found that a decreased MMP-9/TIMP-1 ratio was associated with increased progesterone secretion [49]. Oksjoki et al. [50] found that proα1(IV) collagen and TIMP-3 transcripts were significantly lower in PCOS compared to normal follicular phase ovaries, which accompanied altered immunohistochemical staining of MMP-9 and TIMP4, suggesting the involvement of the basement membrane in the pathogenesis of PCOS. In animal models, granulosa cell oxidative stress increases MMP-2 and MMP-9 levels, which were found to degrade Poly [ADP-ribose] polymerase 1 (PARP1), resulting in apoptosis, which can cause follicular atresia [51], a characteristic of patients with PCOM PCOS.

In follicular fluid in women with PCOS (*n* = 7), the expression of TIMP-2 was lower, while the activities of MMP-2 and MMP-9 and the expression of TIMP-2 were similar to the controls [29]. In a cohort of women classified as overweight with PCOS (*n* = 20), Liu, Guan, and Zheng [24] observed elevated serum levels of MMP-2 and decreased levels of IGFBP-1 compared to the age- and BMI-matched controls, with IGFBP-1 negatively correlating with MMP-2 and insulin levels and with BMI in subjects with PCOS. However, the authors did not examine other MMPs or their TIMP inhibitors. In a similar sized cohort of women classified as obese with PCOS (*n* = 23), Lewandowski, Komorowski, O’Callaghan, Tan, Chen, Prelevi and Randeva [28] observed higher serum levels of MMP-2, MMP-9, and TIMP-1 but not TIMP-2. Diamanti-Kandarakis et al. reported lower serum levels of neutrophil gelatinase-associated lipocalin (NGAL) and MMP-9/NGAL, suggesting lower levels of MMP-9 in women with PCOS compared to the age- and BMI-matched controls, both in the lean and overweight PCOS cohorts. In a larger PCOS cohort (*n* = 80), while no differences in MMP-2, MMP-9, or TIMP-1 levels were observed, TIMP-2 levels were decreased and MMP-2/TIMP-2 and MMP-9/TIMP-1 ratios were increased in the subjects with PCOS compared to the controls [27]. The authors also observed correlations between testosterone and TIMP-2 (negative) and the MMP-9/TIMP-1 ratio (positive) [27]. More recently, Ranjbaran, Farimani, Tavilani, Ghorbani, Karimi, Poormonsefi, and Khodadadi [22] reported no difference in the serum levels of MMP-2, MMP-9, TIMP-1, and TIMP-2 but reported that the gelatinase activity of MMP-9 as well as the MMP-9/TIMP-1 ratio were significantly higher in women with PCOS. In non-obese women with PCOS (*n* = 24), there was no difference in MMP levels, including MMP-2 and MMP-9, compared to their weight- and aged-matched non-obese non-insulin-resistant controls [52]. The variations in the results may be a reflection of differences in the hormonal features of subjects as well as sample sizes and demographics given that the MMP levels appear to be impacted by BMI, IR, and age. In addition, most published studies had smaller samples sizes compared to the PCOS cohort in this study.

In this study, whilst MMP-9 was increased, MMP-2 was decreased, which may suggest that the reduction in MMP-2 may be of benefit to offset the increase in MMP-9. However, both MMP-9 and MMP-2 are involved in menstruation, and their dysregulation has been associated with abnormal uterine bleeding [53] that may contribute to the irregular periods seen in PCOS cases. One of the mechanisms through which MMP-2 may exert its effects is via the degradation of collagen that then allows for the expression of integrins and their binding [54]. This may be of importance as direct integrin binding to IGF-1 is shown to be important for IGF-1 signalling in the ECM [55]. Here, we report that both IGF-1 and its binding protein IGFBP-1 were reduced, in accordance with other studies [23], and that their levels were BMI-dependent in PCOS.

In vitro, CRP increases MMP-2 expression and activity through transcriptional and post-transcriptional mechanisms in human vascular smooth muscle cells [56]. Increase in both CRP and MMP-9 are seen in response to an underlying insult of inflammation, and they are both shown to be inhibited through the same mechanism via the ER-p38/ERK1/2-PPARγ-NF-κB-CRP/MMP-9 signal pathway [57]. In this study, CRP levels were higher in the subjects with PCOS compared to the controls. While there are conflicting reports on increased levels of CRP, a measure of low-grade chronic inflammation in PCOS, a meta-analysis indicates that CRP levels are moderately elevated in PCOS [58]. However, neither MMP-2 nor MMP-9 was associated with CRP, but CRP was associated with TIMP-2, suggesting that the underlying mechanism leading to elevated MMP-9 levels may be due inflammation affecting the inhibitory proteins. MMP-9/TIMP ratios are specifically influenced by weight, with the suggestion that the ratios may be biomarkers for obesity-related diseases such as cardiovascular disease and metabolic syndrome [59], which are conditions associated with PCOS.

We also observed decreased MMP-14 levels and altered MMP-17/TIMP ratios in the subjects with PCOS compared to the controls. MMP-14 and MMP-17 are members of the MT-MMPs and mediators of pericellular proteolysis, modulating proliferation, apoptosis, differentiation, and migration [45]. MMP-14 is a type I transmembrane enzyme with collagenase activity and can activate proMMP-2 unlike the glycosylphosphatidylinositol-anchored protease MMP-17 [45]. MMP-14 may be regulated by the circadian clock, and it controls the rhythmic synthesis of collagen fibres as well as regulates collagen crosslinking [60]. MMP-14 is also related to cancer formation and progression, and its downregulation may be of importance in the endometrium [61], which may be in accordance with the findings reported here, where it was decreased in PCOS and was BMI-dependent; however, together with the changes seen for MMP-9 and MMP-2, it may contribute to the changes in type 4 collagen, suggesting basement membrane alterations in the pathogenesis of PCOS [50].

MMP-17 (MT4-MMP) is associated with inflammation and angiogenesis and is related to the progression of various cancers [62]. Although MMP-17 has limited ECM-degrading abilities compared to other MMPs, exhibiting activity only against gelatine, fibrin, and fibrinogen 1, it is effectively inhibited by both TIMP-1 and TIMP-2 [63]. Whilst MMP-17 did not differ between the subjects with PCOS and controls, the MMP-17/TIMP-1 was decreased in the subjects with PCOS, whilst MMP-17/TIMP-2 was increased, suggesting that there is differential control of MMP-17 depending on the binding of its inhibitors, but it is not clear whether the binding of the different TIMPs to MMP-17 may alter its function. MMP-17 is reported to be expressed in the endometrium in a cycle-dependent manner that corresponds with high angiogenic activity [64], and the altered ratios reported here may contribute to the angiogenic dysregulation associated with PCOS [65].

As previously stated, the serum levels of IGFBP-1 were markedly lower in the PCOS group. In fact, both IGF-1 and its binding protein IGFBP-1 were reduced in PCOS, in accordance with other studies [23], and the IGFBP-1 levels were BMI-dependent in PCOS. In a systematic review and meta-analysis of several heterogeneous studies, IGF-1 was found to be elevated overall in PCOS. However, the elevations were seen in subjects with normal and overweight PCOS and not in subjects with obese PCOS [66] (which is in accordance with the findings in this study, where the mean BMI was 33 kg/m^2^). Li et al. concluded that IGF-1 is BMI-dependent, which is in accordance with the findings presented here that when BMI was accounted for, then IGF-1 did not differ from the controls. IR did not correlate with the MMPs, ADAMS, ADAMTS, or IGF-1/IGFBP-1 when BMI was accounted for, suggesting that it is obesity which is accompanied by IR, rather than IR alone, that may modulate these ECM parameters in PCOS. Whilst androgens did not correlate with the MMPs, ADAMS, ADAMTS, or IGF-1/IGFBP-1 in these subjects with PCOS, androgens may modulate MMPs in other clinical conditions, such as prostate cancer [67].

Adipose tissue accumulation is commonly observed in subjects with PCOS and is worsened by hyperandrogenism [19]. Lifestyle management, specifically weight loss of 5–10% of body weight through regular exercise, is the primary treatment recommendation for women with PCOS [68]. While weight loss has been proven to be successful [69], it can be difficult to sustain, resulting in relapses of weight gain [70], and it is unknown whether MMP-related risk factors are improved by this intervention. In women with PCOS who have undertaken bariatric surgery as a means of weight management, marked reductions in BMI, IR, and androgen levels with a return of regular menses have been observed, coinciding with reductions in both MMP-2 and MMP-9, with the latter being related to changes in adiponectin [71]. Therefore, the current evidence would suggest that early and sustained lifestyle changes may have a long-term beneficial effect in reducing MMP-2 and MMP-9 levels and, at the very least, would not be harmful.

The strengths of this study lie in the large cohort of subjects with PCOS and the measurement of a range of metalloproteases, their inhibitors, and associated proteins. The limitations of this study include that it was carried out on a cohort who were of Caucasian ethnicity. Therefore, the results reported here may not be transmissible to other ethnicities given that the frequency and severity of PCOS phenotypes differ based on race and ethnicity [72]. ADAM-17 [73] and ADAMTS-9 [74] have been reported to play a role in PCOS, but unfortunately, they were not available for analysis in the Soma panel, and they warrant further investigation in this cohort. IR and obesity are highly correlated with PCOS, so adjusting for these confounding variables is challenging given that regression adjustment for either or both likely reduces any inherent PCOS effects. To determine whether a decrease in MMP-related risk factors is dependent on obesity and/or IR would therefore require a matched population of subjects with PCOS that are not obese and not insulin resistant. The Somascan analysis does not identify whether the proteins are active or not, and therefore, one of the limitations of this study is that the results should be treated with a degree of caution until a future functional measurement of MMP forms can be confirmed.

In conclusion, MMP changes in PCOS are largely driven by BMI, though the increased MMP-9 levels were BMI-independent, suggesting that any deleterious effects of MMP-9 would be potentially exacerbated by a concomitantly increased BMI. The significant increases in the MMP-9/TIMP ratios suggests MMP-9 overactivity in PCOS.

## 4. Materials and Methods

### 4.1. Study Design

In total, this cross-sectional study included 234 Caucasian women [75], comprising 137 women diagnosed with PCOS and 97 healthy, regularly menstruating controls with normal physical examinations who had an absence of polycystic ovary morphology (PCOM), as determined by ultrasound. Non-PCOS women, who were recruited by advert, were age-matched to the patients with PCOS, and all were recruited from the same geographic region and with lower socioeconomic status. Healthy control women were not taking any medications, prescription or otherwise. The cohort was from the PCOS biobank (ISRCTN70196169: 2012–2017), approved by the Newcastle & North Tyneside Ethics Committee, and written informed consent for participation was obtained from all subjects [75]. The PCOS diagnosis was made based on the Rotterdam criteria [76], which includes two of the following: oligo/anovulation, hyperandrogenism (Ferriman–Gallwey score > 8, free androgen index > 4 (local laboratory reference level), total testosterone > 1.5 nmol/L (local laboratory reference level)), or transvaginal ultrasound-diagnosed PCOS. Confounding diagnoses and exclusion criteria for the study were appropriately screened as detailed previously [75]. The demographics of the patients with PCOS and healthy controls are shown in Table 4 [75]. Fasting blood samples were centrifuged at 3500× *g* for 15 min, aliquoted, and stored at −80 °C prior to analyses.

### 4.2. Biochemical Parameters

C-reactive protein (CRP) was measured enzymatically (Synchron LX20 analyzer, Beckman-Coulter, High Wycombe, UK). Serum insulin chemiluminescent immunoassay (DPC Immulite 200 analyser, Euro/DPC, Llanberis UK) and plasma glucose (Synchron LX20 analyser, Beckman-Coulter, High Wycombe, UK) measurements were undertaken to determine insulin resistance (IR). In this study, IR was assessed as the homeostasis model assessment (HOMA) for IR, which was derived from the formula HOMA-IR = (insulin × glucose)/22.5. An immunometric assay with fluorescence detection (DPC Immulite 200 analyser, Euro/DPC, Llanberis UK) was employed for measurement of sex hormone-binding globulin (SHBG). Serum testosterone was quantified using isotope dilution liquid chromatography tandem mass spectrometry (LC-MS/MS)(Acquity UPLC system coupled to a Quattro Premier XE mass spectrometer, Waters, Manchester, UK), and the formula ((Total Testosterone/SHBG) × 100) was applied to obtain the free androgen index (FAI) [75].

### 4.3. Metalloprotease and Inhibitor Measurement

Plasma metalloproteases and their inhibitors and related proteins, including IGF-1 and IGFBP-1, were quantified by the Slow Off-rate Modified Aptamer (SOMAmer)-scan platform (version 3.1) (Somalogic, Boulder, CO, USA), which utilises protein–SOMAmer complexes for protein capture on streptavidin beads and an initial biotin-labelled SOMAmer capture, which is then followed by biotin-labelled protein capture [77]. Plasma samples collected in EDTA were processed according to the manufacturer’s instructions as described previously [78,79]. The proteins measured included twelve MMPs, two ADAMS (9 and 12), five ADAMTS (1, 4, 5, 13, and 15), three metalloprotease inhibitors TIMP (1, 2, and 3), IGFBP-1, and IGF-1. Raw intensities, hybridization, median, and calibration signals were normalised and standardised [77,80].

The interactions between the MMPs, ADAMs, ADAMTS, TIMPS, and IGF-1 were examined using the STRINGS 12.0 application.

### 4.4. Statistics

Continuous variables were expressed as means ± standard deviations (SD). Differences between the PCOS and control groups for MMPs, TIMPs, MMP/TIMP ratios, IGF-1, and IGFBP-1 were assessed using independent two-sample *t*-tests. A *p*-value of less than 0.05 was considered statistically significant. Pearson correlation coefficients (*r*) were calculated to assess the relationships between MMPs, TIMPs, BMI, and other continuous variables. An analysis of covariance (ANCOVA) was employed to adjust for potential confounding effects of body mass index (BMI). Statistical analysis was carried out using RStudio (version 2023.03.0). All statistical tests were two-tailed, and a significance threshold of *p* < 0.05 was applied.

## Figures and Tables

**Table 1:** Mean metalloproteases, inhibitors, and insulin-like growth factors in subjects with PCOS and controls.

Parameter (RFU)	PCOS		Controls		
	Mean	SD	Mean	SD	*p*-Value
MMP-1	840	632	761	640	0.34
MMP-2	6558	1512	7065	1153	0.005
MMP-3	1863	9214	694	441	0.213
MMP-7	1108	578	1091	331	0.787
MMP-8	2294	4937	1555	825	0.146
MMP-9	29,187	12,373	22,064	9434	<0.0001
MMP-10	489	382	503	215	0.754
MMP-12	897	306	954	345	0.181
MMP-13	920	1784	580	277	0.064
MMP-14	1097	674	1577	2690	0.04
MMP-16	763	782	1175	4379	0.265
MMP-17	695	213	664	85	0.173
ADAM-9	848	471	1229	2288	0.051
ADAM-12	567	858	478	241	0.318
ADAMTS-1	326	114	314	87	0.389
ADAMTS-4	136	24	133	39	0.468
ADAMTS-5	247	59	237	62	0.178
ADAMTS-13	3846	929	4013	816	0.15
ADAMTS-15	329	59	601	2359	0.163
TIMP-1	48	8	65	155	0.165
TIMP-2	331	54	370	224	0.048
TIMP-3	1515	1070	1495	1148	0.891
IGF-1	739	155	883	354	<0.0001
IGFBP-1	777	730	1529	1119	<0.0001

MMP, matrix metalloproteinases; ADAMS, A disintegrin and metalloproteinase; ADAMTS, A disintegrin and metalloproteinase with thrombospondin; TIMP, tissue inhibitors of metalloproteinases; IGFBP-1 = insulin-like growth factor binding protein 1; IGF-1 = insulin-like growth factor 1.

**Table 2:** Mean MMP/TIMP ratios showing only significant differences in subjects with PCOS and controls. No differences for other MMPs, ADAMS, and ADAMTS were found.

Ratio	PCOS		Control		
	Mean	SD	Mean	SD	*p*-Value
MMP-9/TIMP-1	631.79	298.16	453.32	207.1	<0.01
MMP-9/TIMP-2	91	42.2	65.37	31.8	<0.01
MMP-9/TIMP-3	25.31	17.06	19.58	12.09	<0.01
MMP-17/TIMP-1	14.85	4.32	13.61	2.95	0.01
MMP-17/TIMP-2	2.15	0.69	1.94	0.42	0.01

SD, standard deviation; MMP, matrix metalloproteinases; ADAMS, A disintegrin and metalloproteinase; ADAMTS, A disintegrin and metalloproteinase with thrombospondin; TIMP, tissue inhibitors of metalloproteinases.

**Table 3:** Correlations of metalloproteinases, their inhibitors, and insulin-like growth factors with body mass index in subjects with PCOS.

	BMI	
Parameter (RFU)	*r*	*p*-Value
MMP-1	0.26	0.0036
MMP-2	−0.5	<0.0001
MMP-3	−0.11	0.2397
MMP-7	0.05	0.5961
MMP-8	−0.08	0.3548
MMP-9	0.33	0.0002
MMP-10	−0.31	0.0004
MMP-12	−0.25	0.0047
MMP-13	0.16	0.0672
MMP-14	−0.13	0.1512
MMP-16	−0.08	0.3569
MMP-17	0.14	0.1212
ADAM-9	−0.2	0.0215
ADAM-12	−0.02	0.8553
ADAMTS-1	0	0.9854
ADAMTS-4	0.16	0.0678
ADAMTS-5	0.22	0.0123
ADAMTS-13	−0.24	0.0067
ADAMTS-15	0.27	0.002
TIMP-1	−0.17	0.0585
TIMP-2	−0.16	0.0653
TIMP-3	0.14	0.1256
IGF-1	−0.11	0.2191
IGFBP-1	−0.62	<0.0001

r, Pearson coefficient; MMP, matrix metalloproteinases; ADAMS, A disintegrin and metalloproteinase; ADAMTS, A disintegrin and metalloproteinase with thrombospondin; TIMP, tissue inhibitors of metalloproteinases.

**Table 4:** Demographics, baseline hormonal parameters, and metabolic parameters of subjects with polycystic ovary syndrome (PCOS) and controls. Data are presented as median (IQR).

	PCOS (*n* = 137)	Controls (*n* = 97)	
	Median (IQR)	Median (IQR)	*p*-Value
Age (years)	27.9 (11.0)	28.5 (11.0)	0.09
BMI (kg/m^2^)	33.0 (9.9)	25.0 (5.7)	<0.0001
Body weight (kg)	93.2 (33.3)	68.9 (20.9)	<0.0001
Insulin (IU/mL)	9.0 (8.0)	5.7 (4.1)	0.001
HOMA-IR	2.6 (2.4)	1.3 (1.1)	<0.005
CRP (mg/L)	3.1 (4.7)	1.0 (1.7)	0.0008
SHBG (nmol/L)	21.0 (26.5)	53.5 (37.0)	0.0003
Testosterone (nmol/L)	1.4 (0.9)	1.0 (0.4)	<0.0001

BMI—body mass index; HOMA-IR—homeostasis model of assessment–insulin resistance; CRP—C-reactive protein; SHBG—sex hormone-binding globulin.

## Data Availability

All the data for this study will be made available upon reasonable request to the corresponding author.
